# Successful management of retropharyngeal hematoma by trans-arterial embolism without intubation

**DOI:** 10.1186/s12245-020-00322-9

**Published:** 2021-01-07

**Authors:** Gaku Sugiura, Hiroyuki Takahashi, Yoshihisa Kodama, Satoshi Nara

**Affiliations:** 1 Emergency and Critical Care Medical Center, Teine Keijinkai Hospital, 1-jo 12-chome 1-40, Maeda, Teine-ku, Sapporo, 006-8555 Japan; 2 Department of Radiology, Teine Keijinkai Hospital, Sapporo, Japan

**Keywords:** Computed tomography angiography, Intubation, Hematoma, Embolization

## Abstract

**Background:**

Retropharyngeal hematoma can cause suffocation if there is delay in securing the airway by intubation. However, there are also concerns about complications that can arise with intubation; it is still unknown which cases do not require intubation.

**Case presentation:**

An 88-year-old woman slipped and was found prone and was transported to the emergency room. She was alert without any stridor. Physical examination revealed a subcutaneous hematoma in the anterior cervical region. Computed tomography revealed a retropharyngeal hematoma. Angiography and computed tomography angiography showed extravasation from the right costocervical trunk. A radiologist performed trans-arterial embolization, and she had an uneventful course without intubation or developing any complication. She became ambulatory on postoperative day 5.

**Conclusion:**

Angiography and computed tomography angiography help in early recognition of extravasation in retropharyngeal hematoma, and trans-arterial embolization can help to avoid intubation and its complications.

## Background

The retropharyngeal space is a broad anatomical space [1]. Between the posterior pharyngeal wall and the vertebral bodies, three potential spaces have been described one of which is the middle space. The middle space is a broad space and confluent with the posterior mediastinum. Considerable asymptomatic bleeding into the retropharyngeal space from venous, arterial, and fracture-associated sources can cause suffocation due to delayed hematoma formation. Bleeding may result from venous, arterial, and fracture-associated. To prevent this, early airway securing, such as through intubation and tracheostomy, has become more common [2, 3]. However, there are concerns about complications that may arise from intubation and tracheostomy, such as ventilator-associated pneumonia (VAP), ventilator-induced lung injury (VILI), and deep vein thrombosis (DVT) because of immobilization. It remains unknown which cases of retropharyngeal hematoma (RPH) do not require intubation. There are some studies regarding trans-arterial embolization (TAE) as a management modality for RPH; however, there is no report that TAE prevents delayed hematoma development and suffocation. Here, we report our experience of avoiding intubation by TAE, along with a literature review.

## Case presentation

**Fig. 1:**
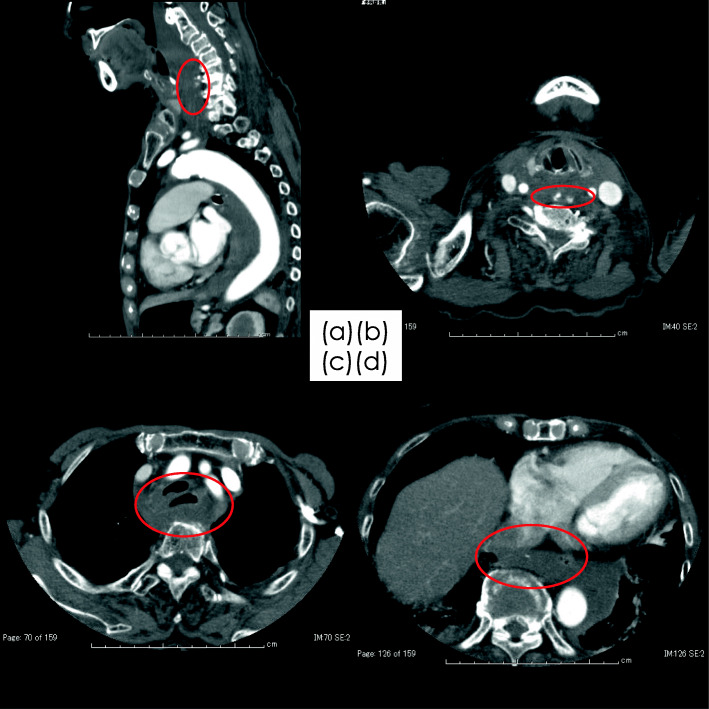
Computed tomography scans show retropharyngeal hematoma and extravasation (circled area) (**a**, **b**). The hematoma extends to the posterior mediastinum and compresses the trachea (**c**, **d**)

**Fig. 2:**
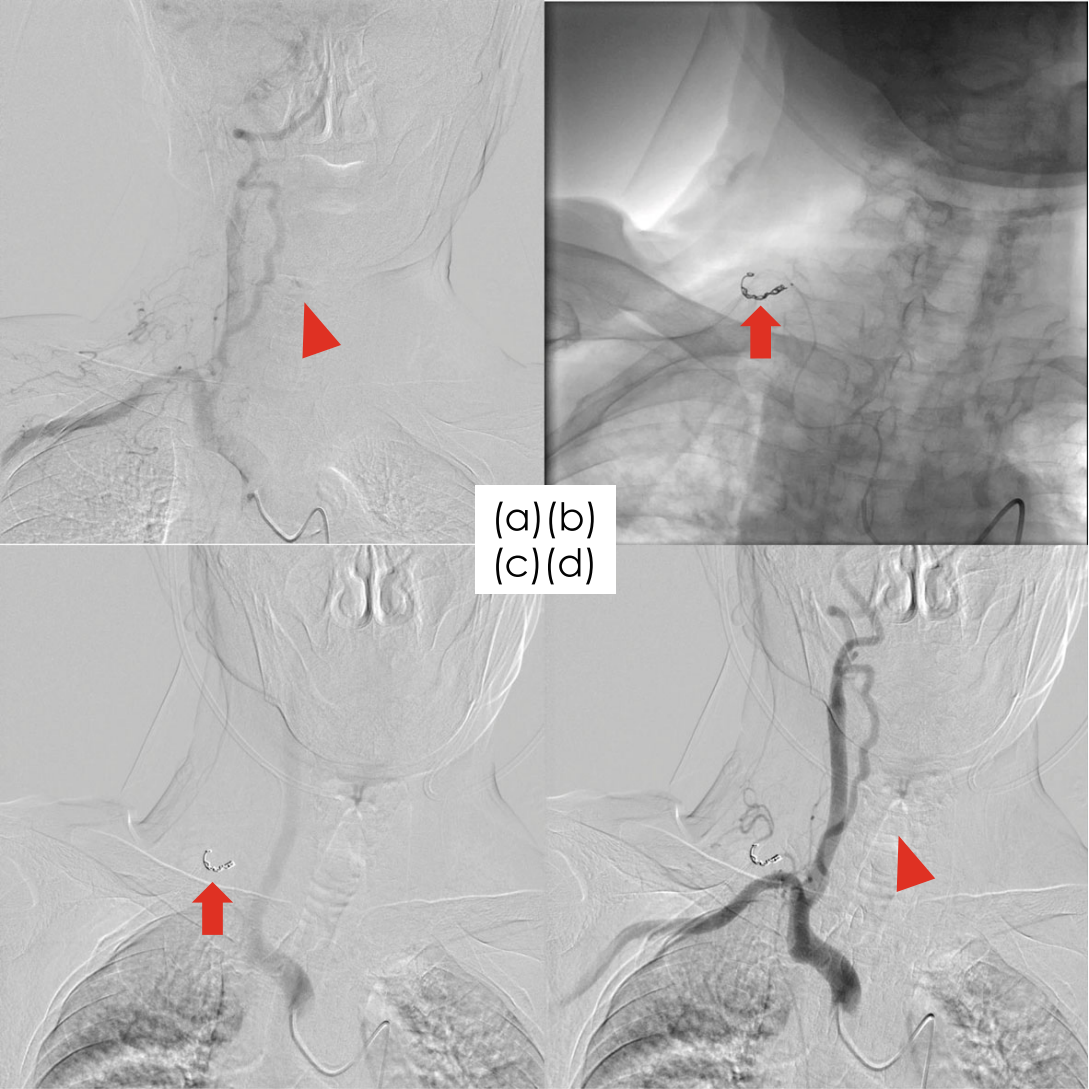
Angiography images show **a** extravasation of contrast media (arrowhead) on the right costocervical artery, **b**, **c** performed TAE (arrow), and **d** the disappearance of the extravasation (arrowhead) after transcatheter arterial embolization

An 88-year-old woman slipped and was found prone in her house by her caregiver and transported to the emergency department by paramedics. She was down for 2 h. Her medical history included thrombophlebitis, and she was taking 2.5 mg warfarin daily. She was alert on presentation. Her vital signs were as follows: respiratory rate, 24 breaths/min; peripheral capillary oxygen saturation was 98% (in room air); blood pressure, 130/60 mmHg; heart rate, 80 b.p.m.; temperature, 36.6 °C. Head and neck examination revealed a subcutaneous hematoma in the anterior cervical region which was 40 mm in diameter, non-pulsatile, not expanding, and not crepitus. Lungs were clear and no stridor at presentation. Neurological, cardiovascular, and abdominal examination were unremarkable. Computed tomography (CT) revealed RPH. CT angiography (CTA) showed extravasation around the hematoma (Fig. 1). To establish hemostasis, an angiography was performed. She was not intubated because she was alert and her respiratory condition was stable. Laboratory analysis revealed that the prothrombin international normalized ratio (PT-INR) was 3.83. We administered 20 mg vitamin K and 1000 IU 4-factor prothrombin complex concentrate (PCC). It also revealed mild anemia and mild thrombocytopenia, but there were no other abnormalities. The radiologist placed 4-Fr-long sheath in the right femoral artery as an access site, thereafter performed coil embolization (Target XL 360®) (Fig. 2) without sedation. She was admitted to the intensive care unit (ICU) with the following vitals: respiratory rate, 17 breaths/min; peripheral capillary oxygen saturation was 99% (in room air); blood pressure, 124/63 mmHg; heart rate, 66 b.p.m.; temperature, 36.5 °C. She was alert. She had an uneventful course without undergoing intubation or developing any complications. On postoperative day 3, she was able to sit in a wheelchair, and she became ambulatory on postoperative day 5. She was downgraded from the ICU on day 12. There was no complication after leaving the ICU. It took time to adjust the referral, and she was transferred to another hospital for rehabilitation on postoperative day 25. Her vitals at time of discharge were as follows: respiratory rate, 15 breaths/min; peripheral capillary oxygen saturation was 97% (in room air); blood pressure, 108/64 mmHg; heart rate, 66 b.p.m.; temperature, 36.0 °C. If dyspnea appeared, she was to refer to a radiologist without follow-up.

## Discussion

RPH is generally associated with cervical spine injury [4]. The thyrocervical artery [4–7] and vertebral artery [1, 8] have been reported as sources of RPH. There are also RPH cases following stellar ganglion block [9] or anterior cervical fusion [10]. Van Velde et al. reported that flexion-extension trauma of the neck without fracture may lead to rupture of the small arteries [5]. This patient did not have cervical spine injury or recent surgery. We consider her mechanism to be similar to that of the previous report. RPH can be diagnosed with plain CT; however, there are limitations to diagnosing arterial bleeding. Therefore, when RPH is confirmed, we recommend CTA in addition. CTA makes it possible to evaluate blood vessels and decide whether to treat them with TAE or other methods, thus obviating intubation. While angiography can be used for evaluation, it carries a 1% risk of stroke when used to evaluate head and neck vessels. Therefore, angiography as a diagnostic procedure has been replaced in recent years by CTA [7].

On literature review, 6 cases were found in which AG was performed for RPH. The literature search was conducted on PubMed. The following keywords were used: “retropharyngeal hematoma” and “retropharyngeal hemorrhage.” Of the 426 abstracts reviewed, 6 cases were selected in which AG was performed (Table 1). In 5 cases, TAE was performed. However, in all cases, TAE was performed after intubation. There have been no reports of avoiding intubation by TAE. In 2 cases, CTA was performed before AG.

**Table 1:** Cases in which AG was performed for retropharyngeal hematoma

S/N	Age (years)	Sex	Reason for intubation	Airway management	Bleeding source	CT	TAE	Outcome	Source, Year
1	83	M	Paradoxical breathing	Endotracheal intubation	Vertebral artery	CTA	y	Limb weakness	Kudo et al., 2017 [8]
2	80	M	Stridor, respiratory distress	Endotracheal intubation	Thyrocervical artery	CECT	y	Not described	Calorego et al., 2015 [6]
3	65	F	Respiratory distress	Nasopharyngeal intubation	Thyrocervical artery	CTA	Coil	Not described	Jakanani et al., 2012 [7]
4	30	F	Stridor, respiratory distress	Endotracheal intubation	Thyrocervical artery	CECT	n	Transferred (day 22)	Iizuka et al., 2012 [4]
5	90	M	Stridor	Endotracheal intubation	Left vertebral artery	CECT	PVA	Extubated (day 3)	Sheah et al., 2006 [1]
6	84	F	Stridor, respiratory distress	Endotracheal intubation	Thyrocervical artery	CECT	PVA	Uneventful	Van Velde et al., 2002 [5]
7	88	F	–	Observation	Costocervical artery	CTA	Coil	Ambulatory (day 5)	This case

*AG* angiography, *CT* computed tomography, *CTA* CT angiography, *CECT* contrast-enhanced CT, *TAE* trans-arterial embolization, *M* male, *F* female, *PVA* polyvinyl alcohol

In this case, selective angiography of the brachiocephalic artery revealed an extravasation in the neck. Bruch of the costocervical artery was considered as the source vessel, and coil was deployed. On literature review, coil and PVA were reported as a modality of embolization. In this case, the radiologist performed coil embolization concerning about cerebral infarction due to embolic substances.

There are also reports suggesting a conservative treatment of RPH with vitamin K and fresh frozen plasma in mild cases with no deterioration of respiratory status [11]. The patient in this case was taking warfarin. Laboratory analysis revealed that PT-INR was prolonged, and the patient was therefore administered vitamin K and a 4-factor PCC. It is important that improving the coagulation state also suppressed bleeding and helped avoid intubation.

Since the patient was alert with intact airway and breathing, she was observed without intubation. In previous reports about acute airway obstruction due to postoperative RPH after anterior cervical fusion, patients without fear, anxiety, and dyspnea could be stabilized with oxygen and close observation [10]. Such close observation is not only monitoring of the respiratory condition but also evaluation of consciousness. If the patient is excited or panicky even without dyspnea, this is a sign of airway obstruction. In this case, the patient was alert without dyspnea, tachypnea, and hypoxemia.

Secondly, by avoiding intubation, respiratory complications, such as VAP and VILI, could be avoided. In addition, intubation invariable immobilizes the patient increasing the risk of DVT. Such immobilization may be prolonged, as some reports indicate that resolution of hematoma needs 2 or 3 weeks, and long-term airway securing is needed [2, 12]. The risk of complications thus increases with long-term airway securing; it is therefore important to perform intubation only in the most necessary cases. In this case, the patient became ambulatory on postoperative day 5 and complications were avoided. On literature review, there are no reports of becoming ambulatory immediately after intubation and AG (Table 1).

TAE is an effective strategy to control arterial hemorrhage, but it cannot control venous hemorrhage and fracture-associated hemorrhage. Therefore, the airway must be promptly secured in the case of venous or fracture-associated hemorrhage. In addition, even if TAE is performed, it is important to monitor the patient closely in the ICU and prepare for intubation if required. An enlarged hematoma can make intubation difficult, and the airway should be secured at the earliest sign of compromise.

## Conclusion

Utilizing CTA in the evaluation of RPH ensures early recognition of extravasation while performing AG and TAE may help to avoid intubation and its possible complications.

## Data Availability

All data is included in the paper and table.
